# Drinking or smoking while breastfeeding and later developmental health outcomes in children

**DOI:** 10.1186/s13104-020-05072-8

**Published:** 2020-04-26

**Authors:** Louisa Gibson, Melanie Porter

**Affiliations:** 1 Faculty of Medicine, Health and Human Sciences, Department of Psychology, Macquarie University, Balaclava Road, North Ryde, Sydney, NSW 2109 Australia; 2 Faculty of Medicine, Health and Human Sciences, Department of Psychology, Macquarie University, Sydney, Australia

**Keywords:** Breastfeeding, Alcohol, Drinking, Tobacco, Smoking, Development, Health, Children, Infants

## Abstract

**Objectives:**

Prenatal intake of alcohol and tobacco have been associated with negative outcomes in children. Consumption of alcohol while breastfeeding has also been associated with dose-dependent decreases in abstract reasoning ability and academic scores in children at later ages. Using longitudinal data from The Growing Up in Australia Study, the current study aimed to investigate whether intake of alcohol or tobacco while breastfeeding was related to later developmental health outcomes in children.

**Results:**

Multivariable linear regression analyses were performed on a sample of 2008 babies who were actively breastfeeding at study entry and 4679 babies who had been breastfed at any time (actively breastfed babies combined with babies who had been previously breastfed). Only a diagnosis of Autism spectrum disorder and Attention deficit disorder were associated with lower developmental health outcomes. Neither maternal alcohol consumption nor tobacco smoking while breastfeeding were associated with developmental health outcomes at 6–7 years old or 10–11 years old for either sample group. A relationship between maternal consumption of alcohol or tobacco smoking while breastfeeding and later developmental health outcomes in children was not identified.

## Introduction

Both alcohol [1] and nicotine [2] pass quickly through to breastmilk. The concentration of alcohol in breastmilk is similar to maternal blood alcohol concentration (BAC) [1]. Nicotine concentration in breastmilk may be higher than maternal BAC [2], and both alcohol and nicotine reduce milk production [3, 4]. Nicotine is also associated with changes in breastmilk composition and taste [3], which may further impact infant feeding and nutritional intake.

Smoking during lactation has been associated with dose-dependent reductions in human milk iodine content [5]. Maternal smoking may also be related to lower birth weight [6], and earlier weaning [7]. Furthermore, prenatal exposure to tobacco smoke has been associated with an increased risk of Attention Deficit/Hyperactivity Disorder (ADHD) both individually, and incrementally in children who have ADHD risk gene variants [8].

An infant’s sleeping and feeding patterns may be disrupted by maternal alcohol consumption during lactation [9, 10]. A case study also described an infant who developed a Pseudo-Cushing syndrome following high alcohol consumption of the mother while breastfeeding [11]. Although Little et al. [12] found reduced psychomotor scores at 12 months of age in babies whose mothers drank while breastfeeding, later studies have not confirmed this association [13, 14].

Dose-dependent reductions in abstract reasoning ability have also been observed in children aged 6–7 years following maternal use of alcohol while breastfeeding [15]. Further analyses found a similar dose-dependent relationship between maternal alcohol consumption during lactation and academic outcomes [16]. This suggests that maternal alcohol consumption while breastfeeding may dose-dependently reduce abstract reasoning ability and academic achievement in children at later ages.

This study aimed to assess whether drinking alcohol or smoking cigarettes during lactation adversely impacts the physical, emotional, social, and school functioning of children. It was hypothesised that alcohol and nicotine use would dose-dependently lower these developmental health scores, independent of pregnancy use.

## Main text

### Method

#### Study design, data source and study cohort

The study design, data source and cohort have been previously described [15, 16]. Briefly, data was sourced from Growing Up in Australia: The Longitudinal Study of Australian Children (LSAC) [17]. The current study comprised 5107 infants and caregivers from LSAC who were recruited in 2004 and followed over time every 2 years in data waves. Wave 1 represents study entry, and six data waves were available for analyses [18, 19]. Only outcomes from waves 4 (age 6–7 years) and 6 (age 10–11 years) were assessed to follow previous studies and compare findings [15, 16]. Further recruitment details are available in LSAC Technical Paper No1 [20].

#### Breastfeeding

Breastfeeding status has been described previously [15, 16]. Briefly, there were two groups of breastfeeding babies. The first group comprised babies who were breastfeeding at Wave 1 (study entry). The second group combined babies who were breastfeeding at Wave 1 with babies who had been breastfed prior to Wave 1, but had stopped by the time they entered the study. Each group was analysed separately.

#### Predictor variables

A modified version of the alcohol use disorders identification test (AUDIT) Alcohol Consumption Questions (AUDIT-C) [21, 22] was given to mothers at Wave 1. A full description of this measure has been previously reported [15]. At Wave 1, mothers were also asked how many days per week they had consumed alcohol during each trimester of their pregnancy, as well as the average quantity they had consumed on each occasion. The number of cigarettes mothers smoked on average per day at Wave 1, and during pregnancy, were used as the measures of tobacco smoking. Further details have been previously reported [15, 16].

#### Outcome variables

Developmental health outcomes were measured using the Pediatric Quality of Life Inventory (PedsQL) Generic Core Scales [23]. The PedsQL are a series of age-specific 23-item measurement tools designed to quantify core indexes of health (physical, emotional and social functioning) as identified by the World Health Organization, as well as school functioning. A total PedsQL score was calculated as the mean across all questions. Scores ranged from 0 to 100, with higher scores indicating better health-related quality of life [23]. The PedsQL Parent Report for Young Children (ages 5–7 years) Acute Version was administered at Wave 4, and the PedsQL Parent Report for Children (ages 8–12 years) Acute Version was administered at Wave 6. Full copies of PedsQL scales are available via the eProvide website [24].

#### Control variables

Details regarding control variables have been described previously [15]. Briefly, they included sex, child age, maternal age, combined family income, maternal education, birthweight, and breastfeeding duration, since these have all been associated with cognitive or academic outcomes in children [25–30]. The diagnosis of Attention Deficit Disorder (ADD) (sic)/ADHD or Autism Spectrum Disorder (ASD; Table 1) was included as a control variable at each wave, since both have been related to impaired social functioning [31]. Attention Deficit Disorder is not a recognised disorder [32], however, LSAC may have included it in the wording of the question to improve communication to caregivers who were not familiar with correct terminology. As described previously [16], in analyses of babies who had been breastfed at any time, breastfeeding status (active or prior) was added as a control variable to account for non-contemporaneous measurement of maternal modified AUDIT-C scores and maternal smoking for infants who had ceased breastfeeding at the time of study entry.

**Table 1:** Descriptive statistics for ASD and ADD (sic)/ADHD variables prior to MI

Variable	Response	N (%)
Does child have any of these ongoing conditions? Autism, Aspergers, or other autism spectrum (sic) (Wave 4)	No	4132 (80.9)
	Yes	107 (2.1)
	Missing	868 (17.0)
Does child have any of these ongoing conditions? Autism, Aspergers, or other autism spectrum (sic) (Wave 6)	No	3551 (69.5)
	Yes	145 (2.8)
	Missing	1411 (27.6)
Does child have any of these ongoing conditions? ADD (sic)/ADHD (Wave 4)	No	4173 (81.7)
	Yes	66 (1.3)
	Missing	868 (17.0)
Does child have any of these ongoing conditions? ADD (sic)/ADHD (Wave 6)	No	3563 (69.8)
	Yes	133 (2.6)
	Missing	1411 (27.6)

#### Statistical analyses

Statistical analyses were identical to that used previously [15, 16], except where specified below. Data was analysed using IBM SPSS version 24. An intention-to-treat type approach was utilised by imputing missing data using multiple imputation (MI). Twenty-eight imputations were used since the highest proportion of missing data for any variable was 28% (Tables 1, 2 and prior [15, 16]). The efficiency and replicability of data is increased by matching the imputation number to missing data percentage when missing data is <50% [33].

**Table 2:** Descriptive statistics for PedsQL scores prior to MI

	Mean (SD)	Range	Missing data N (%)
PedsQL score wave 4	77.94 (13.59)	22.83–100.00	897 (17.6)
PedsQL score wave 6	80.02 (13.66)	15.28–100.00	1444 (28.3)

Multivariable linear regression analyses were performed including all predictor and control variables separately for each outcome variable. The Benjamini–Hochberg procedure [34] was used to correct for Type I error (α = 0.05, 2-tailed).

#### Power analyses

Only data from biological mothers and their children was included. Following MI (d = 0.2, α = 0.05), 99% power was achieved using a pooled sample size of 2008 babies who were breastfeeding at Wave 1 and 16 independent variables. A pooled sample of 4679 babies who had been breastfed at any time provided > 99% power with 17 independent variables [35].

### Results

#### Descriptive statistics (prior to MI)

Descriptive statistics not previously reported [15, 16] are shown in Tables 1, 2.

#### Wave 1 maternal alcohol consumption and tobacco smoking prior to MI

Descriptive statistics relating to maternal alcohol consumption and tobacco smoking have been described previously [15]. Differences in these variables between babies who were actively breastfeeding at Wave 1 and babies who had previously been breastfed have also been outlined [16].

#### Missing data

Little’s Missing Completely at Random (MCAR) test found that data was not MCAR, χ2 = 8164.90, df = 6289, p = <0.0001. Previous analyses have shown that poorly educated parents were more likely to drop out of the LSAC study [36], suggesting data was not missing at random and suitable for MI [37].

#### Babies breastfeeding at wave 1

For Wave 4, the model explained 4–6% of variance across imputations. A diagnosis of ASD or ADD (sic)/ADHD were both associated with lower PedsQL scores. No other statistically significant associations were observed (Additional file 1).

For Wave 6, the model explained 9–12% of variance across imputations. A diagnosis of ASD or ADD (sic)/ADHD were both associated with lower PedsQL scores. No other statistically significant associations were observed (Additional file 2).

#### Babies who had been breastfed at any time

For Wave 4, the model explained 6% of variance across imputations. A diagnosis of ASD or ADD (sic)/ADHD were both associated with lower PedsQL scores. No other statistically significant associations were observed (Additional file 3).

For Wave 6, the model explained 8–9% of variance across imputations. A diagnosis of ASD or ADD (sic)/ADHD were both associated with lower PedsQL scores. No other statistically significant associations were observed (Additional file 4).

## Discussion

Only a diagnosis of ASD or ADD (sic)/ADHD were associated with lower PedsQL scores in both groups and at both time points. Maternal alcohol consumption and tobacco smoking while breastfeeding were not associated with developmental health outcomes in either sample group or time point. Similarly, alcohol and tobacco use during pregnancy, child’s age, sex and birthweight, mother’s age and education, combined family income, breastfeeding duration, and breastfeeding group were not related to PedsQL scores.

The finding that ASD and ADD (sic)/ADHD were associated with lower PedsQL scores is consistent with prior research. Children with ASD and ADHD have both been shown to have poorer social skills [31]. Given that social skills is one of the core indexes of the PedsQL [23], decreased social functioning would also lower overall PedsQL scores.

While no direct comparison exists, the lack of association between breastfeeding alcohol and tobacco use and developmental health outcomes is somewhat consistent with prior research. Although associations between maternal use of alcohol while breastfeeding and cognitive [15] and academic [16] outcomes in children have been identified, associations with basic infant developmental outcomes have been mixed [12–14]. Since developmental screening tools incorporate aspects of physical, emotional and social functioning, it is possible that developmental screens and the PedsQL measure some similar constructs, albeit at different stages of childhood development. The current study supports the finding that maternal use of alcohol and tobacco while breastfeeding does not uniquely impact developmental outcomes.

## Conclusions

Maternal use of alcohol or tobacco were not related to PedsQL scores at either age or in either sample group. This is somewhat consistent with the mixed findings of previous studies assessing infant developmental scores [12–14], suggesting that maternal alcohol or tobacco used while lactating are not associated with unique developmental health outcomes in children. A diagnosis of ASD or ADD (sic)/ADHD was associated with reduced developmental health outcomes in children at ages 6–7 years and 10–11 years in both sample groups. This is likely to be related to the PedsQL incorporating measures of social cognition as a core index [23]. Future studies should to seek to measure alcohol and tobacco intake contemporaneously as well as the timing of alcohol consumption relative to breastfeeding. The scope of research assessing potential relationships between maternal use of alcohol and tobacco while breastfeeding on children at later ages should also be expanded to incorporate a wider range of cognitive and health outcomes. Given interactions between prenatal tobacco exposure and ADHD [8], it would also be interesting to examine whether exposure to tobacco through breastmilk increases risk of ADHD.

## Limitations

The study has many limitations. Measures of pregnancy alcohol and tobacco use were retrospective and may not be accurate representations of genuine usage. Furthermore, AUDIT-C scores and tobacco smoking were measured at Wave 1 making them contemporaneous in the group of babies who were actively breastfeeding at Wave 1, but not in the sample of infants who had stopped breastfeeding by the time of study entry. This may not have a significant impact on the current study, however, since breastfeeding alcohol and tobacco were not related to PedsQL scores in either of the breastfeeding groups.

Similarly, the timing of alcohol consumption relative to feeding was not measured, and it is not known how much, if any, ethanol was available for consumption by the infant. While this is an important consideration, it should not be assumed that any potential deficits caused by maternal drinking are caused by direct consumption of alcohol by the infant. Since it is known that maternal use of alcohol during lactation can alter infant’s feeding and sleeping patterns [9, 10], consumption of alcohol could indirectly impact infants by reducing their nutritional intake, or by altering their sleep/wake patterns.

## Supplementary information

**Additional file 1:** Babies being breastfed at Wave 1: Regression analysis Wave 4 PedsQL scores.

**Additional file 2:** Babies being breastfed at Wave 1: Regression analysis Wave 6 PedsQL scores.

**Additional file 3:** Babies breastfed at any time: Regression analysis Wave 4 PedsQL scores.

**Additional file 4:** Babies breastfed at any time: Regression analysis Wave 6 PedsQL scores.

## Data Availability

The dataset analysed during the current study is available by application to LSAC via the instructions available here: https://growingupinaustralia.gov.au/data-and-documentation

## Abbreviations

ADHD: Attention deficit/hyperactivity disorder
LSAC: Growing up in Australia: the longitudinal study of Australian children
AUDIT: The alcohol use disorders identification test
AUDIT-C: The alcohol use disorders identification test alcohol consumption questions
PedsQL: The pediatric quality of life inventory
ADD*: Attention deficit disorder* (Not a recognised disorder)
ASD: Autism spectrum disorder
MI: Multiple imputation
MCAR: Missing completely at random

## References

[1] Kesäniemi YA (1974). Ethanol and acetaldehyde in the milk and peripheral blood of lactating women after ethanol administration. BJOG: Int J Obstet Gynaecol..

[2] Luck W, Nau H (1984). Nicotine and cotinine concentrations in serum and milk of nursing smokers. Br J Clin Pharmacol.

[3] Napierala M, Mazela J, Merritt TA, Florek E (2016). Tobacco smoking and breastfeeding: effect on the lactation process, breast milk composition and infant development. A Crit Rev Environ Res.

[4] Mennella JA, Pepino MY, Teff KL (2005). Acute alcohol consumption disrupts the hormonal milieu of lactating women. J Clin Endocrinol Metab.

[5] Laurberg P, Nøhr SB, Pedersen KM, Fuglsang E (2004). Iodine nutrition in breast-fed infants is impaired by maternal smoking. J Clin Endocrinol Metabol.

[6] Jaddoe VW, Troe EJW, Hofman A, Mackenbach JP, Moll HA, Steegers EA, Witteman J (2008). Active and passive maternal smoking during pregnancy and the risks of low birthweight and preterm birth: the Generation R Study. Paediatr Perinat Epidemiol.

[7] Horta BL, Kramer MS, Platt RW (2001). Maternal smoking and the risk of early weaning: a meta-analysis. Am J Public Health.

[8] Wang Y, Hu D, Chen W, Xue H, Du Y (2019). Prenatal tobacco exposure modulated the association of genetic variants with diagnosed ADHD and its symptom domain in children: a community based case-control study. Scientific Rep.

[9] Giglia R, Binns C (2006). Alcohol and lactation: a systematic review. Nutr Diet.

[10] Haastrup MB, Pottegard A, Damkier P (2014). Alcohol and breastfeeding. Basic Clin Pharmacol Toxicol.

[11] Binkiewicz A, Robinson MJ, Senior B (1978). Pseudo-Cushing syndrome caused by alcohol in breast milk. J Pediatr.

[12] Little RE, Anderson KW, Ervin CH, Worthington-Roberts B, Clarren SK (1989). Maternal alcohol use during breast-feeding and infant mental and motor development at 1 year. N Engl J Med.

[13] Little RE, Northstone K, Golding J, Team AS (2002). Alcohol, breastfeeding, and development at 18 months. Pediatrics.

[14] Tay RY, Wilson J, McCormack C, Allsop S, Najman JM, Burns L, Elliott EJ, Jacobs S, Olsson CA, Mattick RP (2017). Alcohol consumption by breastfeeding mothers: frequency, correlates and infant outcomes. Drug Alcohol Rev.

[15] Gibson L, Porter M (2018). Drinking or smoking while breastfeeding and later cognition in children. Pediatrics.

[16] Gibson L, Porter M (2020). Drinking or smoking while breastfeeding and later academic outcomes in children. Nutrients.

[17] Growing Up in Australia. publications [http://www.growingupinaustralia.gov.au/pubs/index.html] Accessed 06 Oct 2017.

[18] Australian Institute of Family Studies. The Longitudinal study of australian children: frequently asked questions. [http://www.growingupinaustralia.gov.au/about/faq.html] Accessed 12 Oct 2016.

[19] Australian Institute of Family Studies (2015). Longitudinal study of Australian children data user guide–november 2015.

[20] Soloff C, Lawrence D, Johnstone R. LSAC technical paper no. 1: Sample design. Melbourne, Australia: Australian Institute of Family Studies. 2005.

[21] Babor TF, Higgins-Biddle JC, Saunders JB, Monteiro MG, World Health Organization. AUDIT: The alcohol use disorders identification test: guidelines for use in primary health care. 2001.

[22] Bush K, Kivlahan DR, McDonell MB, Fihn SD, Bradley KA (1998). The AUDIT alcohol consumption questions (AUDIT-C): an effective brief screening test for problem drinking. Arch Intern Med.

[23] Varni JW. PedsQL 4.0: The PedsQL-measurement model for the pediatric quality of life inventory. 1998. https://www.pedsql.org/. Accessed 5 August 2020.

[24] Varni JW. eProvide: PedsQL Review Copies. 1998 [https://eprovide.mapi-trust.org/instruments/pediatric-quality-of-life-inventory/pedsql-review-copies] Accessed 5 August 2020.

[25] Verhaeghen P, Salthouse TA (1997). Meta-analyses of age-cognition relations in adulthood: estimates of linear and nonlinear age effects and structural models. Psychol Bull.

[26] Leigh A, Gong X (2010). Does maternal age affect children’s test scores?. Aust Econ Rev.

[27] Tong S, Baghurst P, Vimpani G, McMichael A (2007). Socioeconomic position, maternal IQ, home environment, and cognitive development. J Pediatr.

[28] Aarnoudse-Moens CSH, Weisglas-Kuperus N, van Goudoever JB, Oosterlaan J (2009). Meta-analysis of neurobehavioral outcomes in very preterm and/or very low birth weight children. Pediatrics.

[29] Stoet G, Geary DC (2013). Sex differences in mathematics and reading achievement are inversely related: within- and across-nation assessment of 10 years of PISA data. PLoS ONE.

[30] Bernard JY, Armand M, Peyre H, Garcia C, Forhan A, De Agostini M, Charles MA, Heude B (2017). Breastfeeding, polyunsaturated fatty acid levels in Colostrum and Child Intelligence Quotient at Age 5–6 Years. J Pediatr.

[31] Bora E, Pantelis C (2016). Meta-analysis of social cognition in attention-deficit/hyperactivity disorder (ADHD): comparison with healthy controls and autistic spectrum disorder. Psychol Med.

[32] American Psychiatric Association (2013). Diagnostic and statistical manual of mental disorders: DSM-5.

[33] Von Hippel P. The number of imputations should increase quadratically with the fraction of missing information. arXiv preprint arXiv:1608.05406. 2016.

[34] Benjamini Y, Hochberg Y (1995). Controlling the false discovery rate: a practical and powerful approach to multiple testing. J Royal Stat Soc Ser B.

[35] Faul F, Erdfelder E, Lang A-G, Buchner A (2007). G* Power 3: a flexible statistical power analysis program for the social, behavioral, and biomedical sciences. Behav Res Methods.

[36] Baxter J: Employment characteristics and transitions of mothers in the longitudinal study of Australian children. Browser download this paper. 2013.

[37] Sterne JA, White IR, Carlin JB, Spratt M, Royston P, Kenward MG, Wood AM, Carpenter JR (2009). Multiple imputation for missing data in epidemiological and clinical research: potential and pitfalls. BMJ.

