# Supplementary material for: Drinking or smoking while breastfeeding and later developmental health outcomes in children
Source: BMC Res Notes. 2020 Apr 26;13:232. doi: 10.1186/s13104-020-05072-8 (PMC7184702; doi:10.1186/s13104-020-05072-8)
Supplement: Supplementary file 1 — Additional file 1: Babies being breastfed at Wave 1: Regression analysis Wave 4 PedsQL scores. [file 13104_2020_5072_MOESM1_ESM.docx]

**Additional file 1**

Babies being breastfed at Wave 1: Regression analysis Wave 4 PedsQL scores.

| **Variable#** | **B Coefficient** | **SE** | **95%CI** | **p value** | **Adjusted p value**** |
| --- | --- | --- | --- | --- | --- |
| Intercept | 131.81 | 11.53 | 109.19-154.43 | ˂0.001 | N/A |
| ASD Wave 4 | -16.82 | 2.34 | -21.41-(-)12.23 | ˂0.001 | ˂0.001 |
| ADD (sic)/ADHD Wave 4 | -10.02 | 3.34 | -16.57-(-)3.47 | ˂0.001 | 0.02 |
| Pregnancy: 2nd trimester days per week drank alcohol | 2.43 | 1.12 | 0.23-4.62 | 0.03 | 0.16 |
| Combined family income* | -0.30 | 0.14 | -0.58-(-)0.01 | 0.04 | 0.16 |
| Pregnancy: Average number of drinks | -1.29 | 0.74 | -2.75-0.16 | 0.08 | 0.26 |
| Pregnancy: 3rd trimester days per week drank alcohol | -1.27 | 0.92 | -3.09-0.54 | 0.17 | 0.45 |
| Pregnancy: 1st trimester days per week drank alcohol | -0.83 | 0.75 | -2.30-0.64 | 0.27 | 0.53 |
| Mother’s modified AUDIT-C score Wave 1 | 0.19 | 0.17 | -0.15-0.52 | 0.27 | 0.53 |
| Mother’s age Wave 1 | -0.06 | 0.07 | -0.19-0.07 | 0.37 | 0.64 |
| Mother’s level of education | -0.20 | 0.24 | -0.68-0.27 | 0.40 | 0.64 |
| Child’s age Wave 4 (months) | 0.75 | 1.15 | -1.51-3.02 | 0.51 | 0.72 |
| Child’s sex | -0.41 | 0.66 | -1.71-0.89 | 0.54 | 0.72 |
| Child’s birth weight (grams) | 0.00 | 0.00 | ˂0.001-˂0.001 | 0.86 | 0.99 |
| Breastfeeding duration (days) | 0.00 | 0.00 | ˂0.001-˂0.001 | 0.92 | 0.99 |
| Average daily cigarettes while pregnant | -0.01 | 0.23 | -0.47-0.44 | 0.95 | 0.99 |
| Mother’s Average daily cigarettes Wave 1 | 0.00 | 0.16 | -0.32--0.32 | 0.99 | 0.99 |

#Variance Inflation Factor<10 for all variables; *Higher scores indicate lower income; **Benjamini-Hochberg method; SE=standard error; CI=confidence interval
