# Supplementary material for: Drinking or smoking while breastfeeding and later developmental health outcomes in children
Source: BMC Res Notes. 2020 Apr 26;13:232. doi: 10.1186/s13104-020-05072-8 (PMC7184702; doi:10.1186/s13104-020-05072-8)
Supplement: Supplementary file 2 — Additional file 2: Babies being breastfed at Wave 1: Regression analysis Wave 6 PedsQL scores. [file 13104_2020_5072_MOESM2_ESM.docx]

**Additional file 2**

Babies being breastfed at Wave 1: Regression analysis Wave 6 PedsQL scores.

| **Variable#** | **B Coefficient** | **SE** | **95%CI** | **p value** | **Adjusted p value**** |
| --- | --- | --- | --- | --- | --- |
| Intercept | 78.92 | 12.00 | 55.28-102.56 | ˂0.001 | N/A |
| ASD Wave 6 | -19.71 | 1.73 | -23.10-(-)16.31 | ˂0.001 | ˂0.001 |
| ADD (sic)/ADHD Wave 6 | -7.62 | 1.71 | -10.98-(-)4.27 | ˂0.001 | ˂0.001 |
| Combined family income* | -0.19 | 0.15 | -0.48-0.10 | 0.19 | 0.58 |
| Mother’s age Wave 1 | -0.08 | 0.07 | -0.21-0.05 | 0.22 | 0.58 |
| Child’s birth weight (grams) | 0.00 | 0.00 | ˂0.001-˂0.001 | 0.28 | 0.58 |
| Mother’s average daily cigarettes Wave 1 | 0.15 | 0.14 | -0.14-0.43 | 0.31 | 0.58 |
| Mother’s modified AUDIT-C score Wave 1 | -0.16 | 0.16 | -0.47-0.16 | 0.32 | 0.58 |
| Pregnancy: 3rd trimester days per week drank alcohol | 0.83 | 0.92 | -0.99-2.65 | 0.37 | 0.58 |
| Average daily cigarettes while pregnant | -0.16 | 0.20 | -0.54-0.22 | 0.41 | 0.58 |
| Pregnancy: 2nd trimester days per week drank alcohol | -0.92 | 1.14 | -3.16-1.33 | 0.42 | 0.58 |
| Child’s age Wave 6 (months) | 0.82 | 1.05 | -1.24-2.88 | 0.43 | 0.58 |
| Pregnancy: 1st trimester days per week drank alcohol | 0.66 | 0.84 | -1.01-2.33 | 0.44 | 0.58 |
| Child’s sex | -0.45 | 0.68 | -1.78-0.88 | 0.51 | 0.62 |
| Breastfeeding duration (days) | 0.00 | 0.00 | ˂0.001-˂0.001 | 0.68 | 0.76 |
| Mother’s level of education | 0.09 | 0.24 | -0.38-0.56 | 0.72 | 0.76 |
| Pregnancy: Average number of drinks | -0.18 | 0.71 | -1.58- 1.21 | 0.80 | 0.80 |

#Variance Inflation Factor<10 for all variables; *Higher scores indicate lower income; **Benjamini-Hochberg method; SE=standard error; CI=confidence interval
