# Supplementary material for: Drinking or smoking while breastfeeding and later developmental health outcomes in children
Source: BMC Res Notes. 2020 Apr 26;13:232. doi: 10.1186/s13104-020-05072-8 (PMC7184702; doi:10.1186/s13104-020-05072-8)
Supplement: Supplementary file 3 — Additional file 3: Babies breastfed at any time: Regression analysis Wave 4 PedsQL scores. [file 13104_2020_5072_MOESM3_ESM.docx]

**Additional file 3**

Babies breastfed at any time: Regression analysis Wave 4 PedsQL scores.

| **Variable#** | **B Coefficient** | **SE** | **95%CI** | **p value** | **Adjusted p value**** |
| --- | --- | --- | --- | --- | --- |
| Intercept | 140.52 | 6.73 | 127.32-153.71 | ˂0.001 | N/A |
| ASD Wave 4 | -16.08 | 1.36 | -18.74-(-)13.42 | ˂0.001 | ˂0.001 |
| ADD (sic)/ADHD Wave 4 | -14.42 | 1.77 | -17.88-(-)10.95 | ˂0.001 | ˂0.001 |
| Combined family income* | -0.24 | 0.09 | -0.42-(-)0.05 | 0.01 | 0.07 |
| Currently or previously breastfed at Wave 1 | 0.93 | 0.46 | 0.04-1.82 | 0.04 | 0.17 |
| Pregnancy: Average number of drinks | -0.97 | 0.49 | -1.94-0.00 | 0.05 | 0.17 |
| Pregnancy: 2nd trimester days per week drank alcohol | 1.41 | 0.83 | -0.23-3.04 | 0.09 | 0.26 |
| Mother’s level of education | -0.25 | 0.16 | -0.56-0.06 | 0.12 | 0.29 |
| Pregnancy: 1st trimester days per week drank alcohol | -0.54 | 0.49 | -1.49-0.42 | 0.27 | 0.58 |
| Mother’s modified AUDIT-C score Wave 1 | 0.09 | 0.11 | -0.13-0.31 | 0.41 | 0.72 |
| Child’s sex | -0.33 | 0.44 | -1.20-0.54 | 0.45 | 0.72 |
| Pregnancy: 3rd trimester days per week drank alcohol | -0.45 | 0.69 | -1.82-0.92 | 0.52 | 0.72 |
| Child’s birth weight (grams) | 0.00 | 0.00 | ˂0.001-˂0.001 | 0.55 | 0.72 |
| Mother’s age Wave 1 | -0.02 | 0.04 | -0.11-0.06 | 0.57 | 0.72 |
| Breastfeeding duration (days) | 0.00 | 0.00 | ˂0.001-˂0.001 | 0.62 | 0.72 |
| Mother’s Average daily cigarettes Wave 1 | -0.04 | 0.07 | -0.18- 0.11 | 0.64 | 0.72 |
| Average daily cigarettes while pregnant | 0.01 | 0.10 | -0.18- 0.20 | 0.90 | 0.91 |
| Child’s age Wave 4 (months) | 0.08 | 0.74 | -1.36-1.53 | 0.91 | 0.91 |

#Variance Inflation Factor<10 for all variables; *Higher scores indicate lower income; **Benjamini-Hochberg method; SE=standard error; CI=confidence interval
