# Supplementary material for: Drinking or smoking while breastfeeding and later developmental health outcomes in children
Source: BMC Res Notes. 2020 Apr 26;13:232. doi: 10.1186/s13104-020-05072-8 (PMC7184702; doi:10.1186/s13104-020-05072-8)
Supplement: Supplementary file 4 — Additional file 4: Babies breastfed at any time: Regression analysis Wave 6 PedsQL scores. [file 13104_2020_5072_MOESM4_ESM.docx]

**Additional file 4**

Babies breastfed at any time: Regression analysis Wave 6 PedsQL scores.

| **Variable#** | **B Coefficient** | **SE** | **95%CI** | **p value** | **Adjusted p value**** |
| --- | --- | --- | --- | --- | --- |
| Intercept | 80.23 | 7.34 | 65.82-94.64 | ˂0.001 | N/A |
| ASD Wave 6 | -19.29 | 1.13 | -21.52-(-)17.07 | ˂0.001 | ˂0.001 |
| ADD (sic)/ADHD Wave 6 | -8.20 | 1.22 | -10.59-(-)5.82 | ˂0.001 | ˂0.001 |
| Mother’s modified AUDIT-C score Wave 1 | -0.17 | 0.10 | -0.37-0.02 | 0.08 | 0.48 |
| Pregnancy: 3rd trimester days per week drank alcohol | 0.99 | 0.72 | -0.43-2.41 | 0.17 | 0.73 |
| Pregnancy: 2nd trimester days per week drank alcohol | -0.93 | 0.81 | -2.52-0.65 | 0.25 | 0.84 |
| Mother’s level of education | 0.15 | 0.16 | -0.16-0.45 | 0.35 | 0.85 |
| Pregnancy: 1st trimester days per week drank alcohol | 0.44 | 0.52 | -0.59-1.46 | 0.40 | 0.85 |
| Mother’s average daily cigarettes Wave 1 | 0.05 | 0.07 | -0.08-0.19 | 0.45 | 0.85 |
| Child’s birth weight (grams) | 0.00 | 0.00 | ˂0.001-˂0.001 | 0.52 | 0.85 |
| Combined family income* | -0.06 | 0.10 | -0.25-0.14 | 0.58 | 0.85 |
| Average daily cigarettes while pregnant | -0.05 | 0.09 | -0.23-0.13 | 0.59 | 0.85 |
| Breastfeeding duration (days) | 0.00 | 0.00 | ˂0.001-˂0.001 | 0.60 | 0.85 |
| Mother’s age Wave 1 | -0.02 | 0.04 | -0.10- 0.07 | 0.67 | 0.86 |
| Child’s age Wave 6 (months) | 0.24 | 0.64 | -1.02- 1.49 | 0.71 | 0.86 |
| Child’s sex | -0.11 | 0.47 | -1.03- 0.81 | 0.81 | 0.92 |
| Pregnancy: Average number of drinks | -0.08 | 0.55 | -1.17- 1.01 | 0.88 | 0.94 |
| Currently or previously breastfed at Wave 1 | -0.03 | 0.49 | -1.00- 0.94 | 0.95 | 0.95 |

#Variance Inflation Factor<10 for all variables; *Higher scores indicate lower income; **Benjamini-Hochberg method; SE=standard error; CI=confidence interval
